# National Cohort Study of Suicidality and Violent Criminality among Danish Immigrants

**DOI:** 10.1371/journal.pone.0131915

**Published:** 2015-06-29

**Authors:** Roger T. Webb, Sussie Antonsen, Pearl L. H. Mok, Esben Agerbo, Carsten B. Pedersen

**Affiliations:** 1 Centre for Mental Health and Safety, Institute of Brain, Behaviour and Mental Health, University of Manchester, Jean McFarlane Building, Oxford Road, Manchester, United Kingdom; 2 NCRR—National Centre for Register-based Research, Aarhus University, Aarhus, Denmark; 3 CIRRAU—Centre for Integrated Register-based Research, Aarhus University, Aarhus, Denmark; Medical University of Vienna, AUSTRIA

## Abstract

**Background:**

Immigrant populations in western societies have grown in their size and diversity yet evidence is incomplete for their risks of suicidality and criminal violence. We examined these correlated harmful behaviours in a national cohort.

**Aims:**

(i) Compare absolute risk between first and second generation immigrants, foreign-born adoptees and native Danes by plotting cumulative incidence curves to onset of early middle age; (ii) estimate sex-specific relative risks for these immigrant type subgroups vs. native Danes; (iii) examine effect modification by higher vs. lower socio-economic status.

**Methods:**

In a cohort of over two million persons, attempted suicides and violent crimes were investigated using data from multiple interlinked registers. We plotted sex-specific cumulative incidence curves and estimated incidence rate ratios.

**Results:**

In the whole study cohort, 1414 people died by suicide, 46,943 attempted suicide, and 51,344 were convicted of committing a violent crime. Among all immigrant subgroups combined, compared with native Danes, relative risk of attempted suicide was greater in female immigrants (incidence rate ratio, 1.59; 95% confidence interval: CI 1.54-1.64) than in male immigrants (1.26; CI 1.20-1.32), and vice versa for relative risk of violent offending in male immigrants (2.36; CI 2.31-2.42) than in female immigrants (1.74; CI 1.62-1.87). Risk for both adverse outcomes was significantly elevated in virtually every gender-specific immigrant type subgroup examined. Violent crime risk was markedly raised in first generation immigrant males and in the Danish born male children of two immigrant parents. However, male immigrants of lower social status had lower risk of attempted suicide than their native Danish peers.

**Conclusion:**

Young immigrants of both first and second generation status face serious challenges and vulnerabilities that western societies need to urgently address. Relative risk patterns for these adverse outcomes vary greatly between the genders and also by socioeconomic status. This high degree of heterogeneity points to the existence of modifiable factors that are amenable to positive change and a potential for effective intervention.

## Introduction

The immigrant populations of western nations have grown substantially in recent times. In 2013, 50.8 million persons residing in European Union (EU) member states were born in a foreign country, just over 10 percent of the total EU population, and nearly two thirds of these individuals were born beyond EU borders [1]. Approximately 40 million persons living in the United States in 2012 were born abroad, constituting almost 13 percent of the national population [2]. Immigrant populations have also become increasingly diverse [3]. In the context of this evolving landscape we require a better understanding of the impact of psychosocial difficulties and psychological distress [4–6], and racial discrimination and marginalisation [7], and how patterns of risk vary between those born abroad and subsequent generations of immigrants. Intergenerational subgroups among Danish immigrants have recently been examined in relation to the full spectrum of psychiatric disorders [8]. Elevated risks were observed across the spectrum, although specificity of risk by diagnosis varied considerably between the subgroups examined.

Herein we report risks of fatal and nonfatal suicidality and violent criminality among all Danish immigrants followed up from adolescence to early middle age. Self-directed violence and violence towards other people are strongly correlated harmful behaviours [9], they share common determinants such as alcohol misuse [10], impulsivity [11], family difficulties [12], and socioeconomic adversity [13], and their combined economic costs to society are enormous [14]. People with history of criminal violence also have a much greater risk of dying by suicide [15]. Nonetheless these two interrelated outcomes have rarely been examined together in the same cohort of individuals [16, 17]. For example, Knox et al. reported that their suicide prevention intervention for United States Air Force personnel appeared to have beneficial effects on lowering risks of homicide and nonfatal domestic violence as well as suicide [17]. Existing literature on violent criminality among immigrants has originated mainly from the United States, where this issue has been of great concern for many decades [18]. More recent studies have tended to focus on immigrants from Mexico and on the most serious forms of violent crime, including homicide, assault with a weapon, and gang violence [19]. Evidence is needed regarding immigrants in European societies, for more common forms of violent crime, for the differential in violence risk between second and first generation immigrants [20], and for effect modification by gender and socioeconomic factors [21]. Suicidality among immigrant populations has attracted widespread attention in Western Europe [22–24], North America [25–27], Australia [28], and Dubai, United Arab Emirates [29]. However, as with violent offending, more evidence is needed for variation in suicidality risk between generations of immigrants [26, 30, 31] for gender-by-culture interactions [32, 33] and for social determinants [22, 34].

This national Danish study had three aims: 1) To compare absolute risk between first and second generation immigrants, foreign-born adoptees and native Danes by plotting gender- and age-specific cumulative incidence curves to early middle age; 2) To estimate sex-specific relative risks for these immigrant type subgroups versus native Danes; 3) To examine effect modification by higher versus lower socio-economic status. On the basis of findings from previous Danish research [35, 36] we hypothesised elevated risk for both internalised and externalised violence. Specifically, we anticipated observing a greater elevation in risk of attempted suicide in female than male immigrants [6, 33] and the reciprocal pattern by gender for violent criminality [37], and also that relative risks would be greater among second generation than first generation immigrants [19, 20, 30, 31]. This intergenerational difference could be driven by acculturative dissonance and intergenerational conflict [20] or by loss of traditional communal protective factors in the second generation [38]. The study’s novelty lies in its nationwide coverage with longitudinal follow-up, direct comparison between suicidality and violent criminality, and estimation of cumulative incidence curves for these two adverse outcomes.

## Materials and Method

### Delineating the study cohort from the Civil Registration System

The study cohort consisted of N = 2,069,114 persons born between 1^st^ January 1971 and 31^st^ December 2002 and residing in Denmark on their 10^th^ birthday. Since 1968 the Civil Registration System has registered all persons living in Denmark [39]. Among other variables, it captures personal and parental identification numbers, gender, date and place of birth, and continuously updated information on vital status. The unique personal identification number is used in all national registers enabling accurate inter-register linkage.

### Classification of adverse outcomes

Cohort members were linked via their personal identifier to nationwide population-based registers to obtain information on violent offending, suicide attempts and deaths by suicide. Since 1980 the National Crime Register has captured information on all criminal charges according to offence type, judicial verdict and sentence [40]. Our definition of interpersonal violent criminality included all convictions for homicide, assault, robbery, aggravated burglary or arson, possessing a weapon in a public place, violent threats, extortion, human trafficking, abduction and kidnapping, rioting, terrorism, and sexual offences (excluding possession of child pornography). The overwhelming majority of these acts consisted of relatively low-level crimes such as assaults, street fights, and threatening behaviour. The study cohort was linked with the Register of Causes of Death [41] to identify suicides using ICD-8 codes 950–959 up to 31^st^ December 1993 [42] and ICD-10 codes X60-X84 from 1^st^ January 1994 [43] by date of death. This Register contains information for all residents who died in Denmark from 1970 onwards.

Cohort members and their parents were linked via their personal identifiers to the Psychiatric Central Research Register [44] and to the National Patient Register [45] to obtain information on suicide attempts, using the exact same classification as was used previously [46]. Identifying these episodes required using different algorithms for different time periods. From 1977 to 1986 they were defined as persons diagnosed with ICD-8 codes E950.0-E959.9 in either the National Patient Register or Psychiatric Central Research Register, from 1987 to 1993 as those admitted with a ‘reason for contact code’ of 4 in the National Patient Register, and after 1994 as fulfilling at least one of the following criteria in either Register:

1. Reason for contact code = 4 (National Patient Register)
2. Any psychiatric diagnosis (ICD-10 chapter F) and a comorbid diagnosis of poisoning with medication and biological compounds (ICD-10 T36 through T50) or non-medical compounds, excluding alcohol and food poisoning (ICD-10 codes T52 through T60)
3. Any psychiatric disorder (ICD-10 Chapter F) and comorbid diagnosis reflecting lesions on forearm, wrist or hand (ICD-10 S51, S55, S59, S61, S65, S69)
4. Any hospital contact due to poisoning with weak or strong analgesics, hypnotics, sedatives psychoactive drugs, anti-epileptics and anti-Parkinsonian drugs or carbon monoxide (ICD-10 T39, T42, T43, and T58)
5. Intentional self-harm: ICD-10 X60-X84 (recorded as a primary or secondary diagnosis in either Register).

### Classification of immigrant status

This status was assigned according to the cohort member and his/her parents’ country of birth as well as mother’s country of residence at the time of the person’s birth. The following subgroups were classified in the same way as previously [8]: inter-country adoptees; first-generation immigrants; second-generation immigrants by one foreign-born parent; second-generation immigrants by two foreign-born parents; native Danes (defined as Danish-born persons with two parents also born in Denmark). The main purpose of deriving these categories was to enable comparison of associations between distinct subgroups with equivalent non-Danish biological parentage: i.e. inter-country adoptees vs. first generation immigrants vs. second generation immigrants by two parents born abroad. Persons who were not classified as above were included in the statistical models, fitted separately in a ‘missing’ category (n = 59,298; 2.9% of the cohort).

### Classification of covariates

Cohort members’ parents were classified as having a history of mental illness if they had received secondary care psychiatric treatment and been diagnosed in ranges ICD-8 290–315 or ICD-10 F00-F99. Socioeconomic status data were obtained from the Integrated Database for Labour Market Research (IDA) [47]. Parental income (quintiles for each year and gender), highest educational attainment level (primary school, high school / vocational training, higher education) and employment status (employed, unemployed, outside workforce for other reasons) were measured during middle childhood, in the year of cohort members’ 10^th^ birthdays. We stratified socioeconomic status using the following algorithm:

- ‘Lower’—Both parents score low in at least one of the three domains: income = lowest quintile; highest education = primary school; employment status = outside the workforce.
- ‘Higher’—Mother and father both employed and score high in at least one of the other two domains: income = highest quintile; education = higher education.
- ‘Middle’—All other combinations.

### Statistical analyses

For examination of suicide and first suicide attempt, individuals were followed up from their 10^th^ birthday until outcome, death, emigration, or December 31^st^ 2011 (for suicide) / December 31^st^ 2012 (for suicide attempt), whichever came first. For first violent crime, individuals were followed up from their 15^th^ birthday until outcome, death, emigration, or December 31^st^ 2011, whichever came first. Gender-specific incidence rate ratios (IRRs) for adverse outcome were estimated by log linear Poisson regression [48, 49], adjusted by age group and calendar year period as time-dependent variables [50]. All other covariates were treated as being time-fixed. P-values and 95% confidence intervals (CIs) were calculated from likelihood ratio tests [50]. Using competing risks survival analyses [51], the cumulative incidence (absolute risk) was calculated as the percentages of persons in the population who had experienced each adverse outcome of interest, taking into account emigration or death from other causes [51, 52]. These analyses were performed separately for each gender and immigration status subgroup. Cumulative incidence at 40^th^ birthday was also calculated for each outcome, separately for males and females.

### Ethics statement

This study was formally scrutinised and approved by the Danish Data Protection Agency, by the Danish State Serum Institute and by Statistics Denmark. It was therefore performed in accordance with the ethical standards laid down in the 1964 Declaration of Helsinki and its later amendments, and in accordance with Danish legislation.

## Results

### Description of the study cohort

All immigrant groups combined represented 14 percent of the 2,069,114 cohort members. In total, including native Danes as well as immigrants, 46,943 people attempted suicide during the follow up period, 1414 died by suicide, and 51,344 were convicted of committing a violent criminal offense, with crude incidence rates of 15.9, 0.5 and 27.1 per 10,000 person-years at risk, respectively. There was a preponderance of females among those who attempted suicide (27,639, 58.9 percent), and the great majority of suicide cases (1136, 80.3 percent) and violent offenders (46,244, 90.1 percent) were males.

### Patterns of risk for all immigrant subgroups combined

We observed a marked gender difference in risk patterns between the two harmful behaviours (Table 1). For female immigrants, relative risks for suicide, attempted suicide and violent offending were of a similar order of magnitude. For male immigrants, relative risk of violent crime was considerably and significantly greater than it was for suicide and attempted suicide. Overall, female immigrants had greater relative risk for attempted suicide than males (gender interaction: P<0.001), whereas male immigrants had a far greater relative risk for violent offending than females (gender interaction: P<0.001). We had insufficient power to assess whether the relative risk for completed suicide was significantly greater in women than in men. We also adjusted the incidence rate ratios for history of secondary care treated parental mental illnesses, but this additional adjustment had a minimal attenuating effect on the strength of the observed associations.

**Table 1:** Incidence rate ratios and cumulative incidences (by 40^th^ birthday) for suicide, attempted suicide and violent criminal offending in the study cohort born 1971–2002: all immigrants combined versus native Danes.

Outcome by immigrant status	Events (n)	Incidence / 10k person yrs.	Incidence rate ratio ^a^ (95% CI)		Cumulative incidence (95% CI)	
**Suicide**						
*Males*:						
Native Danes	1015	0.8	1.00	(ref.)	0.27%	(0.25–0.30)
All immigrants	121	0.8	1.29	(1.05–1.56)	0.26%	(0.21–0.32)
*Females*:						
Native Danes	243	0.2	1.00	(ref.)	0.07%	(0.06–0.08)
All immigrants	35	0.2	1.44	(0.97–2.05)	0.09%	(0.06–0.14)
**Attempted suicide**						
*Males*:						
Native Danes	16,901	12.3	1.00	(ref.)	3.50%	(3.44–3.56)
All immigrants	2403	15.3	1.26	(1.20–1.32)	4.13%	(3.93–4.33)
*Females*:						
Native Danes	22,857	17.9	1.00	(ref.)	4.26%	(4.20–4.32)
All immigrants	4782	31.6	1.59	(1.54–1.64)	6.74%	(6.51–6.98)
**Violent offending**						
*Males*:						
Native Danes	36,737	41.8	1.00	(ref.)	7.28%	(7.20–7.37)
All immigrants	9507	111.5	2.36	(2.31–2.42)	14.83%	(14.47–15.18)
*Females*:						
Native Danes	4179	5.0	1.00	(ref.)	0.90%	(0.86–0.93)
All immigrants	921	10.3	1.74	(1.62–1.87)	1.59%	(1.44–1.76)

^a^ Incidence rate ratio adjusted for age and calendar year period. Further adjustment for secondary care treated parental mental illness had only a minor impact on the observed strengths of association.

### Patterns of risk among immigrant subgroups

**Fig 1:**
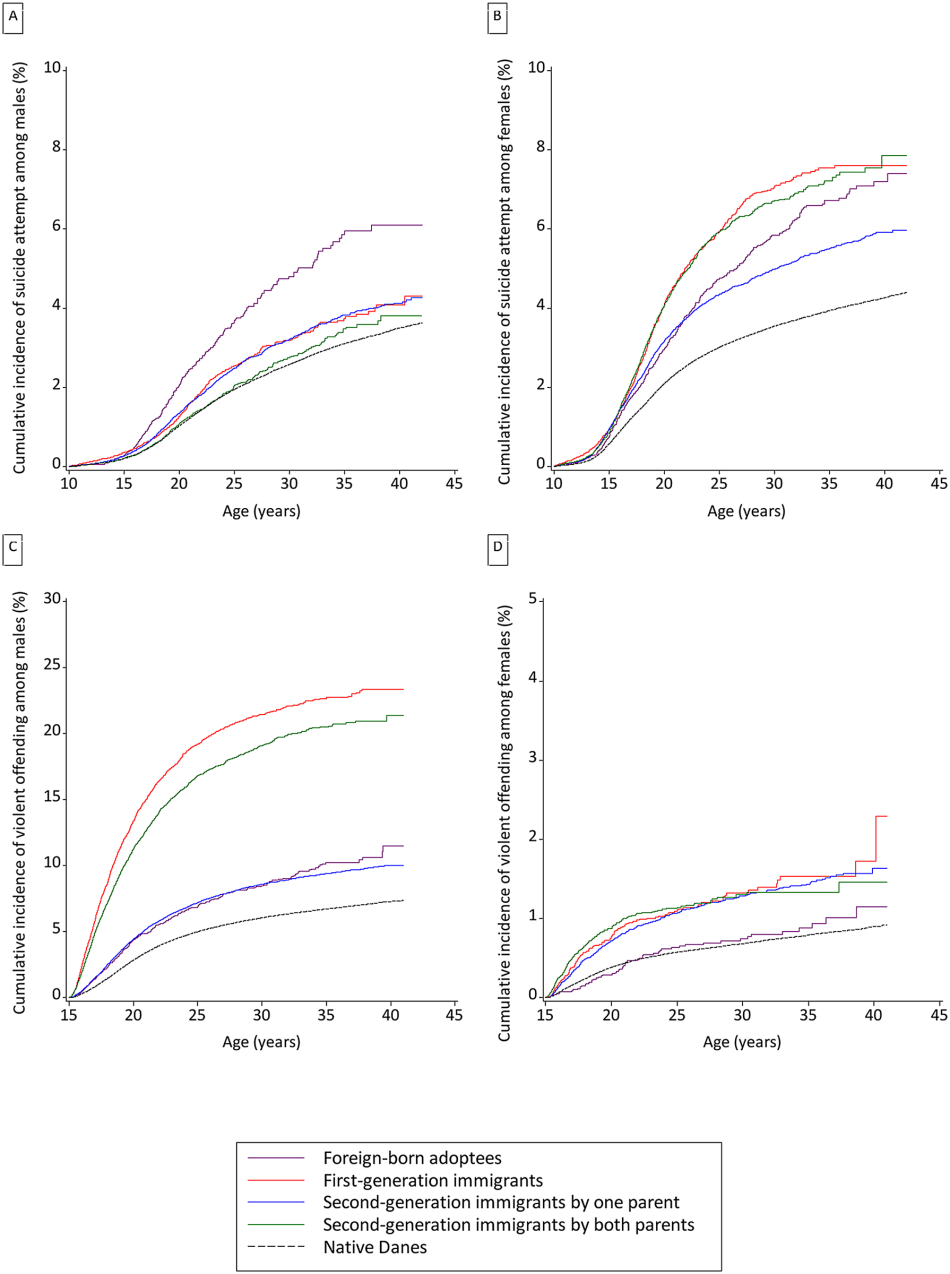
Cumulative incidence curves for attempted suicide and violent offending in the study cohort born 1971–2002: immigrant subgroups and native Danes.

For these finely stratified analyses, we lacked adequate power to examine suicide as an outcome. Cumulative incidence and relative risk for attempted suicide and violent offending, stratified by immigrant subgroup, are presented in Table 2 and in Fig 1 (A-D). Male foreign-born adoptees had a particularly elevated risk of attempted suicide. This group had a 6.1 percent (CI 5.2–7.1) absolute risk of experiencing this outcome before reaching their 40^th^ birthday compared to 3.5 percent (CI 3.4–3.6) in native male Danes. All male immigrant subgroups had a significantly elevated attempted suicide risk except for Danish born children of two immigrant parents, for whom there was no evidence of raised risk. In females the highest cumulative incidences for attempted suicide pre-40^th^ birthday were in second generation immigrants with two foreign-born parents (7.9 percent, CI 7.1–8.7), first generation immigrants (7.6 percent, CI 7.1–8.2) and foreign-born adoptees (7.2 percent, 6.5–8.0). In these groups the cumulative incidences were greater than 7 percent, compared to 4.3 percent (CI 4.2–4.3) among native Danish females. In all four exposure groups, female immigrants had significantly elevated risk for attempted suicide, and the female incidence rate ratios were consistently greater than those for males, except for foreign-born adoptees. The largest female incidence rate ratio was among first generation immigrants.

**Table 2:** Incidence rate ratios and cumulative incidences (by 40^th^ birthday) for attempted suicide and violent criminal offending in the study cohort born 1971–2002: immigrant subgroups versus native Danes.

Outcome by immigrant status	Events (n)	Incidence / 10k person yrs.	Incidence rate ratio ^a^ (95% CI)		Cumulative incidence (95% CI)	
**Attempted suicide**						
*Males*:						
Native Danes	16,901	12.3	1.00	(ref.)	3.50%	(3.44–3.56)
1^st^ generation	411	17.4	1.33	(1.19–1.47)	4.09%	(3.59–4.64)
2^nd^ generation by one parent	1311	15.4	1.27	(1.20–1.35)	4.12%	(3.87–4.39)
2^nd^ generation by both parents	473	11.9	1.03	(0.94–1.13)	3.80%	(3.30–4.36)
Foreign-born adoptees	208	23.1	1.86	(1.61–2.13)	6.10%	(5.21–7.09)
*Females*:						
Native Danes	22,857	17.9	1.00	(ref.)	4.26%	(4.20–4.32)
1^st^ generation	867	41.8	1.95	(1.82–2.10)	7.60%	(7.06–8.18)
2^nd^ generation by one parent	2132	27.1	1.42	(1.35–1.48)	5.91%	(5.62–6.22)
2^nd^ generation by both parents	1335	35.9	1.67	(1.58–1.71)	7.86%	(7.07–8.70)
Foreign-born adoptees	448	30.3	1.71	(1.55–1.87)	7.19%	(6.48–7.95)
**Violent offending**						
*Males*:						
Native Danes	36,737	41.8	1.00	(ref.)	7.28%	(7.20–7.37)
1^st^ generation	2583	212.4	4.25	(4.08–4.43)	23.36%	(22.35–24.38)
2^nd^ generation by one parent	3258	65.5	1.46	(1.41–1.52)	10.03%	(9.63–10.44)
2^nd^ generation by both parents	3307	184.8	3.57	(3.44–3.70)	21.39%	(20.20–22.61)
Foreign-born adoptees	359	65.6	1.47	(1.33–1.63)	11.50%	(9.85–13.29)
*Females*:						
Native Danes	4179	5.0	1.00	(ref.)	0.90%	(0.86–0.93)
1^st^ generation	146	11.8	1.78	(1.50–2.10)	1.73%	(1.29–2.27)
2^nd^ generation by one parent	481	10.0	1.82	(1.65–2.00)	1.63%	(1.43–1.86)
2^nd^ generation by both parents	238	12.7	1.80	(1.57–2.05)	1.46%	(1.17–1.81)
Foreign-born adoptees	56	5.6	1.15	(0.87–1.48)	1.15%	(0.79–1.63)

^a^ Incidence rate ratio adjusted for age and calendar year period. Further adjustment for secondary care treated parental mental illness had only a minor impact on the observed strengths of association.

The highest cumulative risks for violent offending were observed among male first generation immigrants (23.4 percent, CI 22.4–24.4) and in Danish born male children of two immigrant parents (21.4 percent, CI 20.2–22.6), compared to 7.3 percent (CI 7.2–7.4) for native Danes. Modestly raised risks were seen for the other two male immigrant subgroups, although these risk elevations were statistically significant nonetheless. In female immigrants, violent offending risk was raised, but not to the same marked degree as in males. There was a significant elevation in violent criminality risk for all female exposure categories, except for foreign-born adoptees, with incidence rate ratios of 1.8 observed among first and second generation immigrants (by one parent only or both parents). The cumulative incidences of violent offending by early middle age lay in the range 1–2 percent for all the female immigrant subgroups examined.

### Effect modification by socioeconomic status

**Fig 2:**
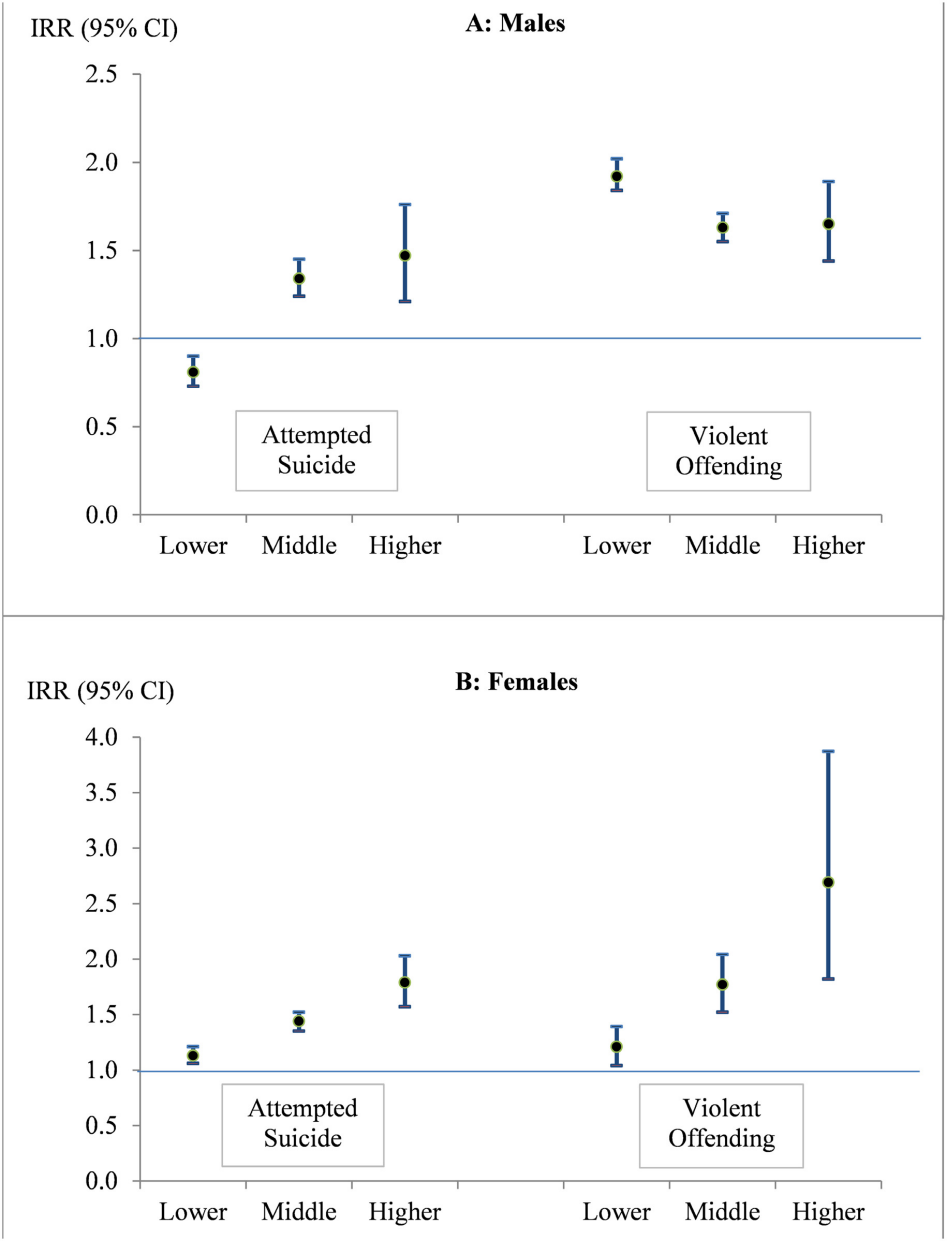
Incidence rate ratios for attempted suicide and violent offending stratified by socioeconomic status in the study cohort born 1971–2002: all immigrants combined versus native Danes. ^a^ Stratification by SES level was made according to parental income, educational attainment and employment status (as is described in detail in the Methods section, p7).

Among males, all immigrants combined had a 2.36 fold increased risk of violent offending compared to native Danes (Table 1). We stratified this effect by socioeconomic status and found that within each stratum male immigrants had raised risk of violent offending, with the greatest elevation observed in the lower stratum (Fig 2A). Attempted suicide risk was raised among male immigrants of middle and higher socioeconomic status, but in the lower socioeconomic group risk was significantly lower versus native Danish males. In female immigrants attempted suicide and violent offending risks were elevated within each stratum (Fig 2B). For both outcomes there was a stepwise increase in the magnitude of the observed relative risk, with the smallest being for the lower socioeconomic stratum and the largest for the higher one.

Table 3 contains detailed gender-specific stratification of relative risk by socioeconomic status according to immigrant status subgroup. Some subgroups contained small event counts, but it reveals a marked heterogeneity by immigrant subgroup that is masked in Fig 2. For instance, the relatively low attempted suicide risk among all male immigrants combined was found only in the first generation and in the Danish born children of two immigrant parents. This apparent protective effect was not seen in male second generation immigrants with only one parent born abroad or in male foreign-born adoptees. Among male first generation immigrants and Danish born male children of two immigrant parents, however, there was a consistent pattern of significantly elevated violent offending risk across all three socioeconomic status strata.

**Table 3:** Incidence rate ratios for attempted suicide and violent offending stratified by socioeconomic status (SES) in the study cohort born 1971–2002: immigrant subgroups vs. native Danes.

Outcome by immigrant status	‘Lower’ SES: IRR (95% CI) ^a^		‘Middle’ SES: IRR (95% CI) ^a^		‘Higher’ SES: IRR (95% CI) ^a^	
**1. Attempted suicide:**						
*Males*:						
Native Danes	1.00	(ref.)	1.00	(ref.)	1.00	(ref.)
1^st^ generation	**0.69**	**(0.55–0.87)**	1.17	(0.78–1.69)	2.39	(0.74–5.57)
2^nd^ generation by one parent	1.10	(0.96–1.25)	**1.31**	**(1.19–1.44)**	0.93	(0.71–1.20)
2^nd^ generation by both parents	**0.51**	**(0.41–0.62)**	1.04	(0.82–1.30)	1.53	(0.66–2.97)
Foreign-born adoptees	1.33	(0.84–1.98)	**2.07**	**(1.68–2.52)**	**3.93**	**(2.94–5.14)**
*Females*:						
Native Danes	1.00	(ref.)	1.00	(ref.)	1.00	(ref.)
1^st^ generation	0.98	(0.85–1.14)	**1.53**	**(1.18–1.95)**	0.67	(0.11–2.06)
2^nd^ generation by one parent	**1.40**	**(1.27–1.55)**	**1.34**	**(1.24–1.45)**	**1.34**	**(1.13–1.59)**
2^nd^ generation by both parents	0.99	(0.89–1.10)	**1.25**	**(1.07–1.45)**	1.34	(0.72–2.25)
Foreign-born adoptees	1.02	(0.71–1.41)	**2.06**	**(1.81–2.34)**	**3.35**	**(2.77–4.02)**
**2. Violent offending:**						
*Males*:						
Native Danes	1.00	(ref.)	1.00	(ref.)	1.00	(ref.)
1^st^ generation	**2.68**	**(2.47–2.90)**	**2.52**	**(2.10–2.99)**	**3.45**	**(1.73–6.07)**
2^nd^ generation by one parent	**1.33**	**(1.23–1.44)**	**1.35**	**(1.26–1.43)**	1.04	(0.85–1.25)
2^nd^ generation by both parents	**2.36**	**(2.20–2.52)**	**2.72**	**(2.46–3.00)**	**1.87**	**(1.05–3.04)**
Foreign-born adoptees	0.82	(0.56–1.14)	**1.73**	**(1.48–1.99)**	**4.34**	**(3.53–5.28)**
*Females*:						
Native Danes	1.00	(ref.)	1.00	(ref.)	1.00	(ref.)
1^st^ generation	0.83	(0.58–1.14)	**2.13**	**(1.14–3.58)**	-	-
2^nd^ generation by one parent	**1.75**	**(1.44–2.11)**	**1.77**	**(1.48–2.10)**	1.62	(0.94–2.81)
2^nd^ generation by both parents	0.96	(0.74–1.22)	**1.81**	**(1.23–2.54)**	3.19	(0.79–12.89)
Foreign-born adoptees	0.64	(0.23–1.38)	**1.60**	**(1.07–2.27)**	**5.67**	**(3.47–9.28)**

^a^ Incidence rate ratio adjusted for age and calendar year period. In this table only statistically significant IRRs are highlighted using bold text. Further adjustment for secondary care treated parental mental illness had only a minor impact on the observed strengths of association

When considering these markedly heterogeneous patterns of risk, it is important to consider differences in socioeconomic status distribution between the immigrant subgroups. Thus, according to the subgroup denominators, more than four-fifths of first generation immigrants, and between two-thirds and three-quarters of second generation immigrants by both parents, were of lower socioeconomic status, compared with less than a half of second generation immigrants by one parent only and less than a third of foreign-born adoptees. (These additional results are not shown, but are available on request from the authors).

## Discussion

### Summary of main findings

Suicidality and violent criminality have not previously been examined and reported jointly in a national immigrant cohort. Our cumulative incidence plots and incidence rate ratio estimates indicate that risks for self-directed versus externalised violence vary greatly between immigrant subgroups as well as for immigrants versus native Danes. Combining all immigrants together we observed distinctly differential patterns of risk by gender. Thus, in female immigrants, relative risk of attempted and completed suicide and of violent offending were of similar magnitude, whereas in male immigrants the relative risk for violent offending was much higher than for attempted or completed suicide. The overall effect size for attempted suicide was larger in females than in males, and it was considerably larger for violent offending in males than in females. When we examined specific immigrant subgroups we observed marked heterogeneity. Thus, in men the highest attempted suicide risk was in foreign-born adoptees, whereas in women they were in first generation immigrants, foreign-born adoptees and second generation immigrants with two parents born abroad. Risks for both harmful behaviours were significantly elevated in all immigrant type by gender strata examined, except for attempted suicide in second generation males with both parents born abroad and violent offending in female foreign-born adoptees. In men, particularly large risk elevations for violent offending were seen in first generation immigrants and among Danish born children of two immigrant parents. Socioeconomic status modified the observed associations considerably. Among all female immigrants, relative risk increased in a gradient across the lower, medium and higher socioeconomic strata for both attempted suicide and violent offending. In male immigrants, effect modification was also present, although the pattern of modification was variable.

### Interpretation of findings and comparison with existing evidence

The larger elevation in attempted suicide risk that we found among female immigrants, and the reciprocal pattern of greater relative risk of violent offending in male immigrations, were anticipated. However, some of our other findings were unexpected. We hypothesised higher risks amongst second compared to first generation immigrants, yet this was not observed with either outcome. In fact first generation immigrants had the largest female incidence rate ratio for attempted suicide, and the largest male incidence rate ratio for violent criminality. Nonetheless we did find elevated risks in second generation immigrants versus native Danes, which may be partly explained by acculturative stress [53] and dissonance [20]. Thus, second generation youths may experience differential rates of integration compared with their parents, sometimes leading to intergenerational conflict and alienation.

Investigators in the United States have reported considerably lower risk of violent offending among recently arrived immigrants versus the native population. This apparent protective effect diminished with increasing length of time spent living in the United States, and this ‘negative assimilation’ effect was also observed among second generation immigrants [19]. In Denmark we found greatly elevated violent offending risk in first and second generation immigrant males. That our findings differed so greatly from what has been found previously in the United States could perhaps be explained by considerable variation in baseline risk of violent crime and by differing historic contexts between the two countries. Thus, the United States has a long history of urban ghettoisation and consequent gang violence, oftentimes delineated strongly along ethnic lines [54, 55]. By contrast these phenomena are relatively new to the Nordic countries, which have absorbed large numbers of immigrants for the first time only during the most recent decades. Emerging evidence from Denmark [56, 57] and elsewhere in Scandinavia [58] suggests that territorial marginalisation of young male immigrants could play an important role, via the development of violent gang cultures to accumulate symbolic ‘street capital’ [58]. Being more likely to have violence inflicted upon them by others in hostile discriminatory environments [59] may also influence some immigrant victims to become perpetrators. Finally, Danish residents of foreign background, young males in particular, may be more likely to have their criminal behaviour detected and be apprehended for it [60]. This could arise should they attract disproportionately close attention from law enforcement agencies. In the United Kingdom, for instance, this phenomenon has been substantiated by the Scarman and Lawrence inquiries into ‘Stop and Search’ policing tactics applied to young men of African-Caribbean descent [61].

These dynamic societal processes, in a country that historically has a relatively low prevalence of street violence and criminal gangs, could perhaps explain the large elevation in violent criminality risk that we found in Danish male immigrants. There are, however, no obvious explanations for the relatively modest increases in violent offending risk we observed in second generation male immigrants with one parent only born abroad versus much higher risk seen in the Danish born children of two immigrant parents. It may be that those with one parent born in Denmark are better adapted to Danish culture than if both parents were born abroad.

It would be interesting to consider elevated risks of suicidality and violent criminality among Danish first generation immigrants in relation to competing social causation and social selection hypotheses [62]. Does post-migration racial discrimination and psychosocial stress cause adverse outcome, or are immigrants more likely to have antecedent predisposing risk factors [63]? Alternatively, positive migratory selection factors could play a salient role, should strongly motivated and resilient individuals be overrepresented among the immigrant population [64]. We could not answer this causation versus selection conundrum using the available registry data, because they contained no measures of pre-migration factors.

It is noteworthy that the largest elevations in violent offending risk were found in male first generation immigrants and in Danish born males with two immigrant parents born abroad. These two subgroups both have two foreign-born parents. Therefore the similarly strong effect sizes perhaps indicate that direct exposure to immigration itself is not the chief cause of these large risk elevations, and that other factors are likely to explain them. It is also worth speculating about the possible mechanisms that may explain the heterogeneous patterns we observed in the gender-specific incidence rate ratios when stratified by socioeconomic status. Fig 2 showed that male immigrants in the lower socioeconomic stratum had a risk of attempted suicide that was significantly less than in native Danes within that stratum, whereas for violent offending these male immigrants had the highest-stratum specific incidence rate ratio. Table 3 shows that these patterns were essentially restricted to male first generation immigrants and to those with both parents born abroad. Cultural factors may be important determinants here. Many of these male immigrants, or their parents, could have been born in non-European countries where suicidal behaviour is not tolerated [65] or where it is considered to be a sign of weakness or even femininity [32]. These findings may therefore suggest that lower socioeconomic status immigrant males more frequently express distress via externalised violence rather than through harming themselves. Among female immigrants, those in the lower socioeconomic strata had the lowest incidence rate ratios for both adverse outcomes, and the female effect sizes rose in incremental fashion across the socioeconomic strata. This pattern could perhaps be explained by lower socioeconomic status female immigrants, especially those originating from non-western countries, being strongly protected by their parents and other close family members from social stressors and from peers experiencing psychosocial difficulties, right up until the age when they marry [66]. Such close social control and protection may be less likely to occur among young immigrant females in the middle and higher socioeconomic strata.

### Strengths and limitations

Our national epidemiological study was characterised by generic strengths that apply to most Scandinavian register-based epidemiologic investigations, including: complete record linkage between multiple registers; ability to account comprehensively for death or emigration during follow-up; nationwide coverage in the registry datasets; and abundant statistical power and precision for examining relatively rare adverse events in a cohort of more than 2 million persons. Specific strengths of our study included complete country of birth classification for all cohort members and their parents; ability to examine absolute and relative risks for multiple immigrant status subgroups; and complete ascertainment of hospital treated attempted suicide and convictions for violent criminal offending. We could also examine how cumulative incidence increased from older childhood (attempted suicide) and mid-adolescence (criminal offending) up to the early middle age years. This longitudinal approach contrasts with some previous investigations that used more temporally restrictive cross-sectional designs [67, 68].

Our study findings may have questionable applicability to other western nations, specifically those with far longer histories of mass immigration, racially demarked urban segregation, and gang-related violent crime. Other possible generalisability factors concern differences in countries and regions of the world that immigrants originate from, and immigration policy variability. Some countries have highly restrictive immigration policies, which may serve to screen out individuals and families who appear predisposed to adverse outcome. Also, we could not assess whether risks of attempted suicide and violent criminal offending were convergent or divergent compared with these risks in immigrants’ countries of origin. Such studies have been conducted in relation to suicide [69, 70], but equivalent data sources do not exist in Denmark or other western nations to enable such an assessment in relation to more frequently occurring nonfatal outcomes. Restriction of the study cohort to persons living in Denmark on their 10^th^ birthday meant that it could not be representative of all first degree immigrants in Denmark, since many of these individuals arrived in the country at an older age. The impact of this potential selection bias is unknown. Finally, although our study was well-powered for studying attempted suicide and violent criminality, it was insufficiently powered to examine risk of suicide stratified by multiple immigrant subgroups.

## Conclusion

We observed elevated risks for self-directed violence and externalised violence almost universally across all the immigrant subgroups examined, and in both genders. This should greatly concern experts across numerous health and social care domains, including forensic, child & adolescent and adult mental health services, primary care, hospital emergency departments, and social services. It should also interest those working in law enforcement and correctional and probation services, and in urban planning and redevelopment. The variable magnitude of relative risks observed between immigrant subgroups indicates the existence of factors that are amenable to positive change, and therefore a potential for developing effective interventions. For example, during 2012 the Mayor of London and the English capital’s Crime Reduction Board established a Partnership Anti-Gangs Strategy in collaboration with the city’s numerous local municipalities, the National Health Service Commissioning Board, the Metropolitan Police, the Crown Prosecution Service and the London Probation Trust. The focus of this initiative is on developing more effective multi-agency and cross-municipality working and data sharing to prevent young people at risk from being drawn into gangs, to improve levels of community engagement, and to reduce risk of reoffending. Such an approach could be tackled elsewhere, should its effectiveness be demonstrated [71]. There is a lack of interventions to reduce risk of self-harm among ethnic minority populations, but in order to be effective, such initiatives will need to remove barriers that currently prevent these people from seeking help when they encounter psychosocial difficulties [72].

Further research is urgently needed to identify why some male immigrants in Denmark have such an elevated risk of violent criminality. Whatever mechanisms are involved, it seems clear that young immigrants of both first and second generation status face serious challenges and vulnerabilities that western societies need to urgently address, and that relative risk patterns for these adverse outcomes vary greatly between the genders and also by socioeconomic status. It is necessary to understand the degree to which these heterogeneous risk patterns can be generalised to immigrants living in other countries, and researchers should also investigate risk of violence victimisation in this population.
